# Using the Semantic Web for Rapid Integration of WikiPathways with Other Biological Online Data Resources

**DOI:** 10.1371/journal.pcbi.1004989

**Published:** 2016-06-23

**Authors:** Andra Waagmeester, Martina Kutmon, Anders Riutta, Ryan Miller, Egon L. Willighagen, Chris T. Evelo, Alexander R. Pico

**Affiliations:** 1 Department of Bioinformatics - BiGCaT, Maastricht University, Maastricht, The Netherlands; 2 Maastricht Centre for Systems Biology (MaCSBio), Maastricht University, Maastricht, The Netherlands; 3 Gladstone Institutes, San Francisco, California, United States of America; Hellas, GREECE

## Abstract

The diversity of online resources storing biological data in different formats provides a challenge for bioinformaticians to integrate and analyse their biological data. The semantic web provides a standard to facilitate knowledge integration using statements built as triples describing a relation between two objects. WikiPathways, an online collaborative pathway resource, is now available in the semantic web through a SPARQL endpoint at http://sparql.wikipathways.org. Having biological pathways in the semantic web allows rapid integration with data from other resources that contain information about elements present in pathways using SPARQL queries. In order to convert WikiPathways content into meaningful triples we developed two new vocabularies that capture the graphical representation and the pathway logic, respectively. Each gene, protein, and metabolite in a given pathway is defined with a standard set of identifiers to support linking to several other biological resources in the semantic web. WikiPathways triples were loaded into the Open PHACTS discovery platform and are available through its Web API (https://dev.openphacts.org/docs) to be used in various tools for drug development. We combined various semantic web resources with the newly converted WikiPathways content using a variety of SPARQL query types and third-party resources, such as the Open PHACTS API. The ability to use pathway information to form new links across diverse biological data highlights the utility of integrating WikiPathways in the semantic web.

## Author Summary

WikiPathways is a crowd-sourced online platform for biological pathways. It is based on the same underlying platform as Wikipedia. Pathways are saved as graphical images embedded in a set of meta data elements (i.e. references, list of pathways elements, and context annotations). Pathways are used as proxies of biological knowledge in their role as descriptors of processes. Yet integrating these hubs of biological knowledge with other biological data resources remains challenging due to a cacophony of file formats, identifier systems, and hidden content. We show the application of the semantic web to enable a straightforward integration of heterogeneous biological data sources. We have taken high-quality pathways from a curated set from WikiPathways and converted the content into a data format native to the semantic web. Here, data is expressed as a set of statements where the statements are built upon a set of web addresses. *Given the results, we successfully integrated external resources (e.g., EBI Expression Atlas) and pathway content with a single query*.

## Introduction

Pathway analysis and visualisation of data on pathways provide insights into the underlying biology of effects found in genomics, proteomics, and metabolomics experiments [1–4]. WikiPathways is a pathway repository where content is provided by the community at large [5, 6]. In a given pathway, elements like genes, proteins, metabolites, and interactions are identified using common accession numbers from reference databases such as Entrez Gene [7], Ensembl [8], UniProt [9], HMDB [10], ChemSpider [11], PubChem [12] and ChEMBL [13]. Multiple databases can be referenced to annotate an element of the same semantic type, e.g. Ensembl and Entrez Gene to annotate gene information. Even single studies sometimes use different reference databases to annotate experimental findings. It is common for bioinformaticians to spend valuable time dealing with data mapping issues that impede the actual data analysis and interpretation. In WikiPathways we use the open source software framework BridgeDb [14], to help resolve different identifiers representing the same (or related) entities. Capturing a semantically correct description of biological entities and their connections across datasets is the broader challenge that we have to address. The semantic web provides an approach to define entities and their relationships. By explicitly defining these entities and relationships the semantic web can provide a network of *linked data* [15]. The Resource Description Framework (RDF) consists of two key components: statements and universal identifiers. Each statement is captured as a *triple*, consisting of a subject, a predicate, and an object. For example, the following triple defines the glucose molecule as being part of the glycolysis pathway:

$$\underset{\text{subject}}{\underset{︸}{<\text{Glycolysis}>}}\underset{\text{predicate}}{\underset{︸}{<\text{Has}\;\text{member}>}}\underset{\text{object}}{\underset{︸}{<\text{Glucose}>}}$$

The notion of a semantic web surfaces as you link across large sets of triples representing a vast number of objects and diverse types of concepts and predicates. The use of uniform identifiers, or URIs [16], provides consistency when specifying subjects and objects. identifiers.org [17], for example, provides a clearinghouse for a wide variety of URIs for biological entities in the life science domain. WikiPathways provides identifiers for all its pathways and identifiers.org provides the URI scheme to make these resolvable. Standardized URIs for predicates come from efforts such as the Simple Knowledge Organization System (SKOS) [18]. For example, our example triple above can be expressed in a more universal way as:

$$\underset{\text{subject}}{\underset{︸}{<\text{http}://\text{identifiers}.\text{org}/\text{wikipathways}/\text{WP}534>}}\underset{\text{predicate}}{\underset{︸}{<\text{http}://\text{www}.\text{w}3.\text{org}/2004/02/\text{skos}/\text{core}\#\text{member}>}}$$

$$\underset{\text{object}}{\underset{︸}{<\text{http}://\text{identifiers}.\text{org}/\text{chebi}/\text{CHEBI}:4167>}}$$

where each element is uniquely and universally resolvable to a defined concept (glycolysis, “has member”, and glucose respectively). Of course, the more human readable information can also be explicitly added by describing the labels in RDF. But that information is also available by resolving the URIs.

PREFIX rdfs: <http://www.w3.org/2000/01/rdf-schema#>
PREFIX wp: <http://identifiers.org/wikipathways/>
PREFIX skos: <http://www.w3.org/2004/02/skos/core#>
PREFIX chebi: <http://identifiers.org/chebi/CHEBI:>
wp:WP534 skos:member chebi:4167.
wp:WP534 rdfs:label “Glycolysis and Gluconeogenesis (Homo sapiens)”@en.
chebi:4167 rdfs:label “Glucose”@en.

In order to contribute pathway knowledge to the semantic web, we have modeled the content of WikiPathways to form triple-based statements. The interactions and reactions curated at WikiPathways are particularly well-suited to enrich the overall connectivity of the semantic web. Pathways offer a meaningful context for relations between biological entities, such as proteins, metabolites and diseases that are otherwise defined in disparate databases. We report on the conversion process and the development of two new vocabularies essential in capturing the semantics behind pathway diagrams. Finally, we evaluate the use of the semantically linked pathway knowledge through specialized queries and third-party resources, showing how to link WikiPathways with disease annotations (from UniProt [9] and DisGeNET [19]), with gene-expression values (from Gene Express Atlas) and with bioactive chemical compounds known to affect proteins that occur in pathways (e.g. from ChEMBL).

## Results and Discussion

### Pathway vocabularies

There are existing standards to model various aspects of pathway knowledge, such as BioPAX [20], SBGN [21], MIM [22], SBML [23] and SBO [24]. BioPAX and SBO are in fact already available in a Semantic Web-compatible language called OWL [25]. These standards provide valuable building blocks for our “WP” vocabulary that captures the biological meaning of pathways. However, not all of the graphical annotations, spatial information and other subtleties critical for the visual representation, the intuitive understanding and the usability for data visualisation of the curated content at WikiPathways are captured by these standards. Our “GPML” vocabulary directly reflects these features defined in the XML format, GPML, or Graphical Pathway Markup Language. For example, in GPML, all genes, proteins and metabolites are types of data nodes, which are rendered as a rectangular box with properties capturing among others its position, height, width, label, and external reference. For example:

<DataNode TextLabel = “Glucose” GraphId = “dba83” Type = “Metabolite”>

 <Graphics CenterX = “279.0” CenterY = “468.0” Width = “112.0” Height = “20.0” ZOrder = “32768”>

 <Xref Database = “ChEBI” ID = “CHEBI:4167” />

</DataNode>

In the GPML vocabulary, used for semantic representation of pathway diagrams, the markup elements and values are described as classes and properties, each with their respective URIs.

<http://identifiers.org/chebi/CHEBI:4167> rdf:type gpml:DataNode.

<http://identifiers.org/chebi/CHEBI:4167> rdfs:label “Glucose”@en.

<http://identifiers.org/chebi/CHEBI:4167> gpml:graphId “dba83”.

<http://identifiers.org/chebi/CHEBI:4167> gpml:ZOrder 32768.

…

The GPML vocabulary, in its current form, is mainly instrumental in the representation of the spatial information captured at WikiPathways. However, as we will describe below it can also be used to convert pathway information from other semantic web resources into a format amenable to being rendered and curated at WikiPathways. Explicit mappings to external (graphical) ontologies are not added, however through plugins such as Pathvisio-MIM [26] mappings to graphical notations such as MIM or SBGN, are possible. In an analogous way, the WP vocabulary can be used to capture the biological relations from other pathways in such a way that they can be used in resources using this semantic layer of the WikiPathways RDF. We used this approach for example to make the relations from Reactome pathways available in the Open PHACTS discovery platform [27] starting from the converted pathways at WikiPathways.

The WP vocabulary, focusing on biological meaning, issues URIs for biological concepts and disregards layout and other rendering details. Using URIs from this vocabulary allows stating that something is a Pathway, or that a DataNode is a chemical compound or gene product. The vocabulary also captures descriptive elements, such as labels, shapes and lines that help annotate and contextualize the pathway reaction details. The RDF generated consist of terms from the vocabularies developed in this context. This is done to be able to reflect the semantics used in the WikiPathways community. However, to allow integration with external pathway resources—which is the primary objective of this project—we need to link to external ontologies. For the subset of concepts in common with prior vocabularies, such as BioPAX, we utilize the SKOS data model to express a range of similarities from skos:exactMatch to skos:closeMatch [18, 28].

### Pathway conversion and queries

**Fig 1:**
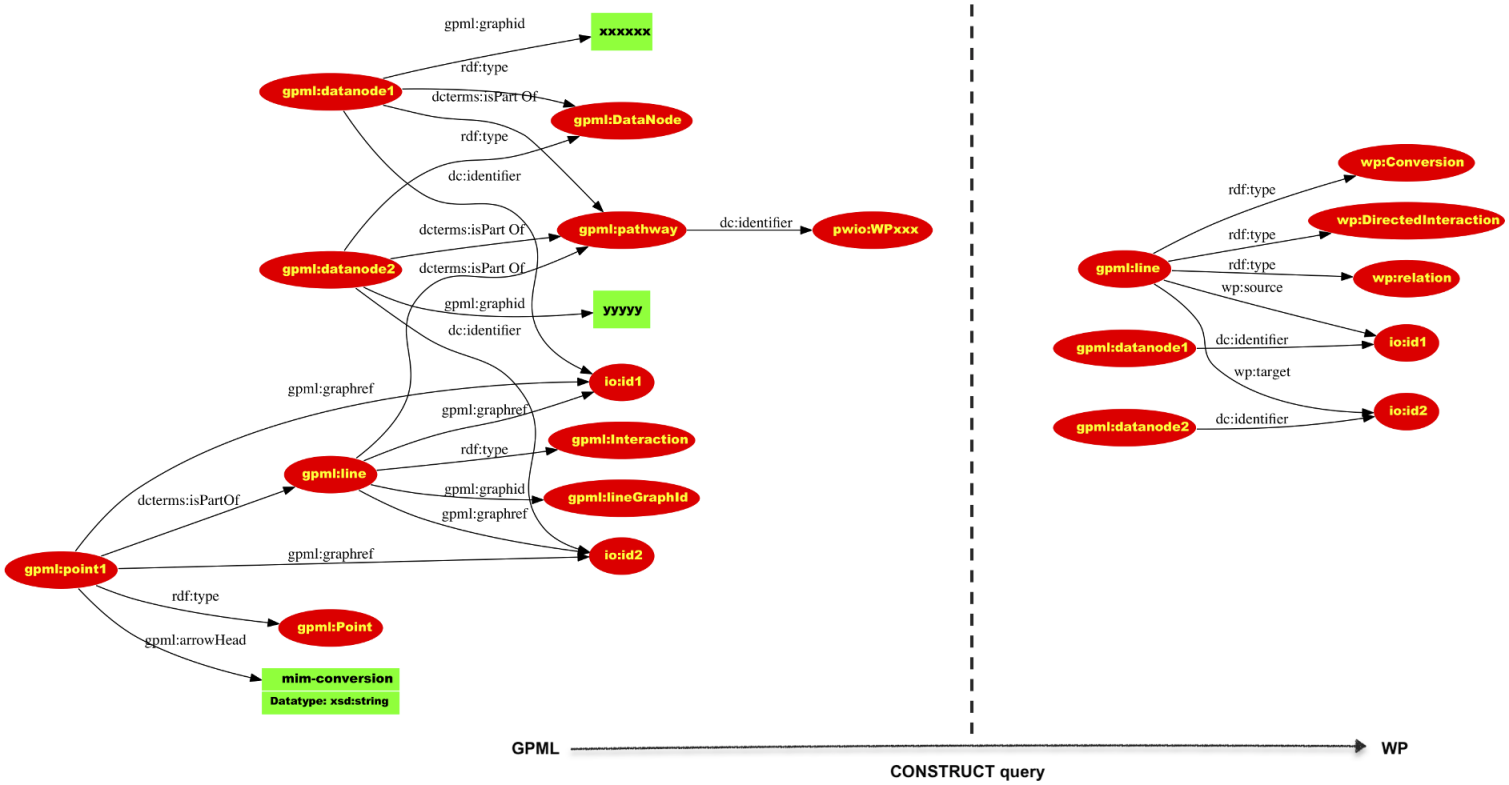
A construct query is type of SPARQL query that enables the conversion of one graph pattern to another. Here an interaction described by its spatial properties (GPML) is converted into a semantic representation reflecting its biological interpretation (WP). The SPARQL query is available in the supporting information section.

With these vocabularies in place, the next step is the actual conversion of GPML files into triples using the GPML vocabulary. Then rules are applied to make the biological meaning explicit using the WP vocabulary. For example a directed interaction is captured in GPML as two “DataNodes”, a line and an arrowhead. The “DataNodes” have external references as properties. Rules are then applied to state that a line is a Directed Interaction, with a source and a target. Fig 1 contains an example of such a rule based reasoning query that issues triples with URIs from the WP vocabulary.

WikiPathways pathways are regularly curated by a team of volunteers that evaluate their usability for analysis and tag the pathways as “curated”. WikiPathways contains 1000 pathways in the curated set across over a dozen species that convert to a total of 1.6 million triples. The triples are loaded in a SPARQL endpoint (http://sparql.wikipathways.org), which allows semantic querying of the data with the SPARQL query language [29]. RDF, including new and updated pathways, is generated and tested regularly and can be delivered upon request. Updates of the RDF that is available for download and in the SPARQL endpoint are triggered by crucial events, such as Reactome or Open PHACTS data releases. This prevents discrepancies in quality control or curation, due to small differences between (frequent) releases. Example SPARQL queries and their plain language translations are given in Table 1. A broad set of ∼50 queries is available on the help pages of WikiPathways [30].

**Table 1:** Example queries handled by the WikiPathways SPARQL endpoint.

List the species captured in WikiPathways and the number of pathways per species	**SELECT DISTINCT** ?organism ?label count(?pathway) as ?numberOfPathways **WHERE** { ?pathway dc:title ?title. ?pathway wp:organism ?organism. ?pathway wp:organismName ?label. ?pathway rdf:type wp:Pathway. } **ORDER BY DESC**(?numberOfPathways)
Get all gene products on a particular pathway (WP615 as an example)	**SELECT DISTINCT** ?pathway ?label **WHERE** { ?geneProduct a wp:GeneProduct. ?geneProduct rdfs:label ?label. ?geneProduct dcterms:isPartOf ?pathway. ?pathway rdf:type wp:Pathway. **FILTER regex**(**str**(?pathway), “WP615”). }
Return all PubChem compounds in WikiPathways and the pathways they are in	**SELECT DISTINCT** ?identifier ?pathway **WHERE** { ?concept dcterms:isPartOf ?pathway. ?concept dc:source “PubChem-compound”^^ xsd: string. ?concept dc:identifier ?identifier. ?pathway rdf:type wp:Pathway }

A *federated* SPARQL query [17] enables querying over multiple SPARQL endpoints. With a variety of SPARQL endpoints available with data on disease annotations (e.g. DisGeNET and UniProt), significantly expressed genes (e.g. EBI Expression Atlas) and drug-target interactions (e.g. ChEMBL), knowledge from these remote SPARQL endpoints can be integrated. Example queries are given in Table 2 and on the help pages of WikiPathways [30]

**Table 2:** Example federated queries handled by the WikiPathways SPARQL endpoint.

From DisGeNET get disease-gene pairs on asthma and get all pathways where these genes have a role
**PREFIX** identifiers: <http://identifiers.org/ensembl/> **PREFIX** atlas: <http://rdf.ebi.ac.uk/resource/atlas/> **PREFIX** efo: <http://www.ebi.ac.uk/efo/> **PREFIX** sio: <http://semanticscience.org/resource/> **PREFIX** skos: <http://www.w3.org/2004/02/skos/core#> **PREFIX** ncit: <http://ncicb.nci.nih.gov/xml/owl/EVS/Thesaurus.owl#> **SELECT DISTINCT** ?wpId ?pwtitle (group_concat(**distinct** ?wpgene_identifier;separator = “; ”) as ?wpgenes) **WHERE** { SERVICE <http://rdf.disgenet.org/sparql/> { **GRAPH** <http://rdf.disgenet.org> { ?gda sio:SIO_000628 ?gene,?disease. ?gene rdf:type ncit:C16612; rdfs:label?geneLabel. ?disease rdf:type ncit:C7057; rdfs:label?diseaseLabel. **FILTER regex**(?diseaseLabel, “asthma”, “i”) ?gene sio:SIO_010078?protein. } } ?wpgene wp:bdbEntrezGene ?gene. ?wpgene dcterms:identifier ?wpgene identifier. ?wpgene dcterms:isPartOf ?pathway. ?pathway a wp:Pathway. ?pathway dc:identifier ?wpId. ?pathway dc:title ?pwtitle. }
For the genes differentially expressed in asthma (found in the EBI Expression Atlas), get the gene products associated to a WikiPathways pathway
**PREFIX** identifiers: <http://identifiers.org/ensembl/> **PREFIX** atlas: <http://rdf.ebi.ac.uk/resource/atlas/> **PREFIX** atlasterms: <http://rdf.ebi.ac.uk/terms/atlas/> **PREFIX** efo: <http://www.ebi.ac.uk/efo/> **SELECT DISTINCT** ?wpURL ?pwTitle ?Ensembl ?EntrezGene ?expressionValue ?pvalue **WHERE** { SERVICE <https://www.ebi.ac.uk/rdf/services/atlas/sparql> { ?factor rdf:type efo:EFO_0000270. ?value atlasterms:hasFactorValue ?factor. ?value atlasterms:isMeasurementOf ?probe. ?value atlasterms:pValue ?pvalue. ?value rdfs:label ?expressionValue. ?probe atlasterms:dbXref ?dbXref. } ?pwElement dcterms:isPartOf ?pathway. ?pathway dc:title ?pwTitle. ?pathway dc:identifier ?wpURL. ?pwElement wp:bdbEnsembl ?Ensembl. ?pwElement wp:bdbEntrezGene ?EntrezGene. } **ORDER BY ASC**(?pvalue)

### Using linked data in common analysis platforms

**Fig 2:**
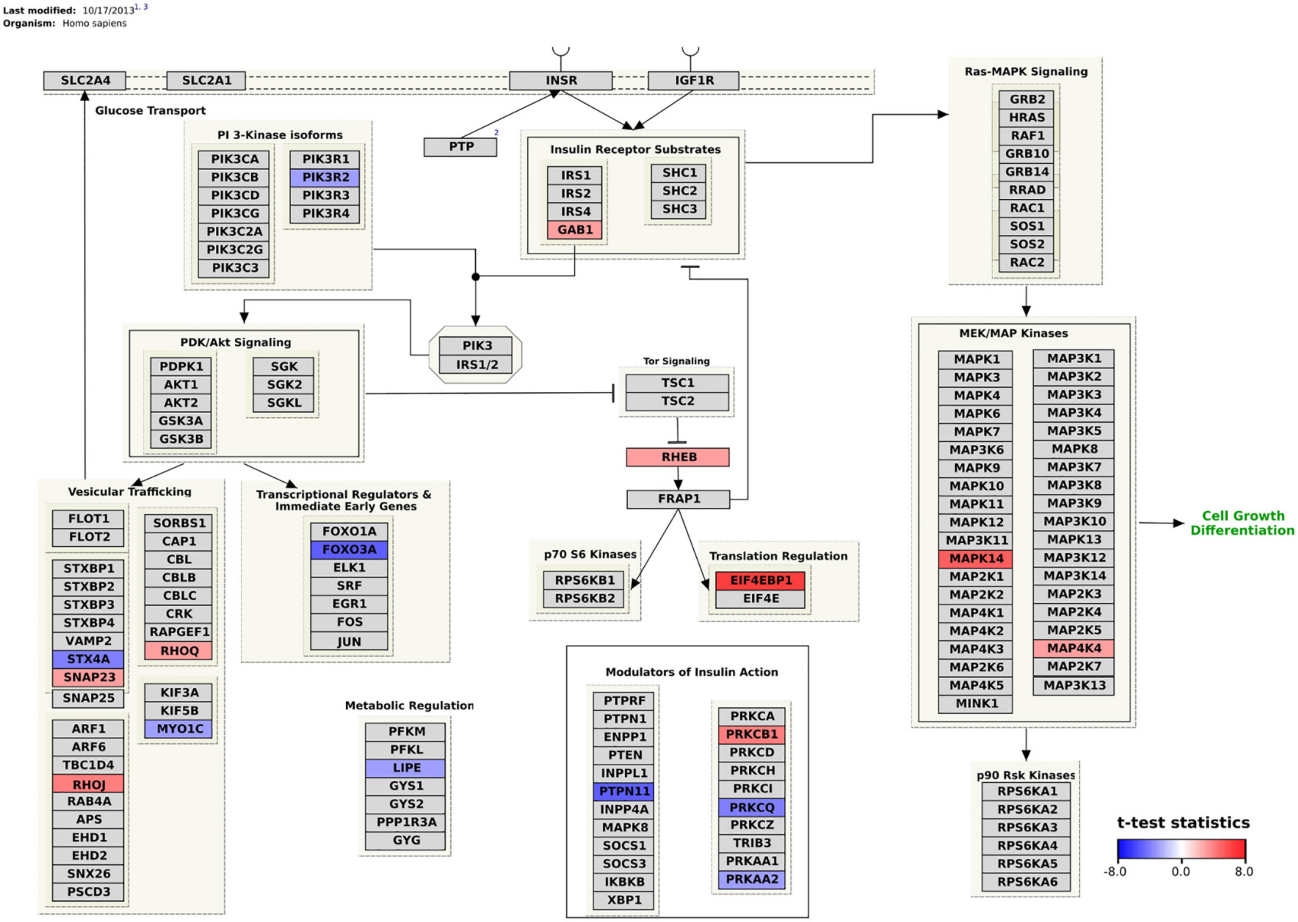
The colored boxes represent genes which are up (red) or down (blue) regulated in diabetes mellitus. PIK3R2, MYO1C, PRKAA2, LIPE are down regulated in pre-diabetes. STX4A is down regulated in type 1 diabetes longstanding. PRKCQ, PTPN11, FOXO3A are down regulated in type 2 diabetes. GAB1, RHEB, MAP4K4, SNAP23 are up regulated in pre-diabetes. RHOJ, PRKCB are up regulated in type 1 diabetes recent onset. MAPK14UP, EIF4EBP1 are up regulated in type 1 diabetes clinical onset. From these 17 up or down regulated genes, 9 are being reported as being in the top 10 disease and phenotype associations for the selected gene in DisGeNET (i.e. PIK3R2, PRKAA2, LIPE, STX4A, PRKCQ, FOXO3A, MAP4K4, SNAP23, and PRKCB) (Gene-disease association data were retrieved from the DisGeNET Database, GRIB/IMIM/UPF Integrative Biomedical Informatics Group, Barcelona. (http://www.disgenet.org/). 04, 2016)

Different common analysis platform allow the integration of linked data for future analysis and visualization. One nice example of such a analysis platform is R, a widely used software environment for statistical computing and graphics. R has a SPARQL library [31], which enables the import of linked data for further processing in R. This allows running common statistical tests or the creation of different visualization of linked data. We recently published an R library that interfaces R with PathVisio [32] and allows manipulation of pathways and data visualisation on pathways. Fig 2 shows up and down regulated genes in Diabetes Mellitus (efo:EFO_0000400, efo:EFO_0001359, and efo:EFO_0001360) in the pathway diagram on insulin signaling in human [30]. This pathway diagram with color-coding parts indicating up- and down regulated pathway elements, was created by integrating knowledge from two geographically dispersed and independent resources, through a single SPARQL query embedded in a R script, which is available online [33].

### Rosetta stone function

A number of resources provide content from multiple pathway databases, including Pathway Commons [34] and NCBIs BioSystems (http://ncbi.org/biosystems). While BioPAX in fact is RDF, the NCBI system is not. NCBI BioSystems uses NCBIs native identifiers: GeneId, ProteinId, CID. We thus have a resource with pathways from different origins that are already described in the same way. Since for WikiPathways content we know how the different entities in these resources map to the GPML and WP vocabularies we can now use that to produce RDF using these same ontologies for each of the other pathway resources present in NCBI BioSystems. In fact, we can do the same for Pathway Commons where this approach will lead to an improved version of RDF with explicit mappings to the WP vocabulary. We made a prototype script available on GitHub to be used for this type of conversions from BioSystems [35].

### Use in discovery platforms

The semantically linked pathway data from WikiPathways RDF have also been integrated into the Open PHACTS discovery platform [27, 36]. Open PHACTS delivers and sustains an open pharmacological space using semantic web standards and technologies. The Open PHACTS platform currently provide 51 API methods of which thirteen deliver pathway information (https://dev.openphacts.org/docs). Other information collected in Open PHACTS describes other relationships like drug-target (from ChEMBL) and protein interaction (from UniProt). Having this all in one resource combined with a set of mapping tools allows fast analysis across the domains. By combining Open PHACTS API calls one can, for instance, find all protein targets for a drug and then all pathways that contain these targets.

## Materials and Methods

### Use of Open PHACTS RDF guidelines

In collaboration with partners in the Open PHACTS project, we proposed guidelines for presenting data as RDF [37], most of that can be considered as general guidelines to produce RDF in the biomedical domain. The guidelines consist of a prerequisite and 11 steps, covering the licensing (step 0), designing (step 1–5), implementation (steps 6–9), and presentation (steps 10–11) of the data in the semantic web. In the work presented here we follow these steps:

#### Licensing

WikiPathways content is covered by the Creative Commons Attribution 3.0 Unported license (https://creativecommons.org/licenses/by/3.0/). This is stated in the VoID headers of the RDF made. These headers are automatically generated by the same script generating the WikiPathways RDF. Open PHACTS provides a template for these header files.

#### Implementation

We used a Java RDF framework, Jena (http://jena.apache.org/)[38], to generate the RDF for WikiPathways. The pathway diagrams were obtained through the web services of WikiPathways, after which they were converted into RDF with the Jena RDF framework. The code of the serializer is available on GitHub (https://github.com/wikipathways/wp2lod). The vocabularies were generated with a vocabulary framework called Deri Neologism (http://neologism.deri.ie/).

#### Presentation

The resulting RDF triples are available from (http://rdf.wikipathways.org) and loaded on a instance of the Virtuoso Open-Source Edition (http://virtuoso.openlinksw.com/) and available through its SPARQL endpoint at http://sparql.wikipathways.org. The triples are also loaded on the Open PHACTS discovery platform (https://dev.openphacts.org/docs/1.5) where they can be accessed through eleven API calls.

### Identifier mapping

In the context of the semantic web, it is impractical to burden query writers with handling identifier mapping per resource and per query. Rather, the mapping results themselves need to become part of the semantic web. We applied two distinct approaches to addressing identifier mapping in our WikiPathways and Open PHACTS projects.

#### Query expansion

The Open PHACTS framework provides query expansion functionality through its Identifier Mappings Services. When an identifier is queried the SPARQL query is enriched with all possible identifiers to retrieve an expanded set of related entities. This approach is the most efficient in terms of the number of triples, since it requires only a single identifier per relationship, eliminating redundancy. However, it also requires a hosted identifier mapping service that it called along with every query.

#### Unified identifiers

In the case of WikiPathways, which does not host a mapping service, we chose a unified identifier approach, where all identifiers are mapped ahead of time to a set of common identifier systems. In this way, the database effectively contains the results of a limited number of identifier mappings in form of partially redundant triples. For example, in the WikiPathways RDF, all identifiers have been unified to Entrez Gene [7] (wp:bdbEntrezGene), Ensembl [8] (wp:bdbEnsembl), UniProt [9] (wp:bdbUniprot) for gene products and HMDB [10] (wp:bdbHmdb), and ChemSpider [11] (wp:bdbChemspider) for compounds like metabolites and drugs. The original identifier provided by the pathway curator is stored as a triple, with the predicate dc:identifier, and a URI from identifiers.org, which points to both the identifier and the resource.

### Summary

We present a semantic web representation of WikiPathways together with vocabularies needed to cover the graphical pathway layout and the biological meaning and solutions to map between different identifier systems. The public availability allows rapid integration with other biological resources. The availability of two vocabularies allows to convert between different pathways resources. Different analytical tools now support the import of semantic web data, allowing integrated use of data from different resources with a single query. We demonstrate this with a federated query across multiple resources where the resulting differentially expressed genes for a disease where shown on a discovered pathway using PathVisio.

### Availability

The following resources are publically available as beta releases just like WikiPathways. They are maintained as part of the open source WikiPathways project

### Vocabularies

- GPML: http://vocabularies.wikipathways.org/gpml
- WP: http://vocabularies.wikipathways.org/wp

### WikiPathways on the Semantic Web

- SPARQL endpoint: http://sparql.wikipathways.org
- Open PHACTS: https://dev.openphacts.org/docs/
- RDF greendownload: http://rdf.wikipathways.org

### Source code

- GitHub: https://github.com/wikipathways/wp2lod

## Supporting Information

S1 File CONSTRUCT query to translate from the GPML vocabulary to the WP vocabulary. A construct query is type of SPARQL query that enables the conversion of one graph pattern to another. Here an interaction described by its spatial properties is converted into a semantic representation reflecting its biological interpretation.
(PDF)
