# Supplementary material for: Using the Semantic Web for Rapid Integration of WikiPathways with Other Biological Online Data Resources
Source: PLoS Comput Biol. 2016 Jun 23;12(6):e1004989. doi: 10.1371/journal.pcbi.1004989 (PMC4918977; doi:10.1371/journal.pcbi.1004989)
Supplement: S1 File — A construct query is type of SPARQL query that enables the conversion of one graph pattern to another. Here an interaction described by its spatial properties is converted into a semantic representation reflecting its biological interpretation. (PDF) [file pcbi.1004989.s001.pdf]

Supplementary Material

S1 Example CONSTRUCT query to translate from the GPML vocabulary to the WP vocabulary

```
# This CONSTRUCT query annotates WikiPathways Directed Interactions.
PREFIX rdf:     <http://www.w3.org/1999/02/22-rdf-syntax-ns#>
PREFIX dc:      <http://purl.org/dc/elements/1.1/>
PREFIX dcterms:  <http://purl.org/dc/terms/>
PREFIX gpml:    <http://vocabularies.wikipathways.org/gpml#>
PREFIX wp:      <http://vocabularies.wikipathways.org/wp#>
PREFIX xsd:     <http://www.w3.org/2001/XMLSchema#>
CONSTRUCT {
        ?line rdf:type wp:Conversion .
        ?line rdf:type wp:DirectedInteraction .
        ?line rdf:type wp:relation .
}
FROM <http://rdf.wikipathways.org/>
WHERE {
        # Get the pathway identifier
        ?pathway dc:identifier ?wpIdentifier .
        # An interaction is between 2 datanodes
        # DataNode 1
        ?datanode1 dc:identifier ?dn1Identifier .
        ?datanode1 gpml:graphid ?dn1GraphId .
        ?datanode1 rdf:type gpml:DataNode .
        ?datanode1 dcterms:isPartOf ?pathway .
        # DataNode 2
        ?datanode2 dc:identifier ?dn2Identifier .
        ?datanode2 rdf:type gpml:DataNode .
        ?datanode2 dcterms:isPartOf ?pathway .
        ?datanode2 gpml:graphid ?dn2GraphId .
        # Some DataNodes don't contain an identifier
        FILTER (!regex(str(?datanode2), "noIdentifier")) .
        FILTER (!regex(str(?datanode1), "noIdentifier")) .
        # The base of an interaction is the line of type gpml:
            ↪ Interaction
        ?line rdf:type gpml:Interaction .
        ?line dcterms:isPartOf ?pathway .
        ?line gpml:graphid ?lineGraphId .
        # A line is linked to a DataNodes by it graphref.
        ?line gpml:graphref ?dn1GraphId .
        ?line gpml:graphref ?dn2GraphId .
        FILTER (?datanode2 != ?datanode1)
        # Directionality is captured in the Points attached to
        # a line. Since both datanodes can be a target of a
            ↪ direction
        # we need to use a UNION to capture both
        ?point rdf:type gpml:Point .
        ?point dcterms:isPartOf ?line .
        ?point gpml:arrowHead "mim-conversion"^^xsd:string .
        {{?point gpml:graphref ?dn2GraphId .} UNION {?point gpml:
            ↪ graphref ?dn1GraphId}}.
        }
```

Figure 1. A construct query is type of SPARQL query that enables the conversion of one graph pattern to another. Here an interaction described by its spatial properties is converted into a semantic representation reflecting its biological interpretation.
